# Semantic Interaction Meta-Learning Based on Patch Matching Metric

**DOI:** 10.3390/s24175620

**Published:** 2024-08-30

**Authors:** Baoguo Wei, Xinyu Wang, Yuetong Su, Yue Zhang, Lixin Li

**Affiliations:** School of Electronic Information, Northwestern Polytechnical University, Xi’an 710129, China; xw18765@outlook.com (X.W.); syt15332639458@mail.nwpu.edu.cn (Y.S.); zhangyue662020@163.com (Y.Z.); lilixin@nwpu.edu.cn (L.L.)

**Keywords:** meta-learning, few-shot learning, supervision collapse, semantic interaction, patch matching

## Abstract

Metric-based meta-learning methods have demonstrated remarkable success in the domain of few-shot image classification. However, their performance is significantly contingent upon the choice of metric and the feature representation for the support classes. Current approaches, which predominantly rely on holistic image features, may inadvertently disregard critical details necessary for novel tasks, a phenomenon known as “supervision collapse”. Moreover, relying solely on visual features to characterize support classes can prove to be insufficient, particularly in scenarios involving limited sample sizes. In this paper, we introduce an innovative framework named Patch Matching Metric-based Semantic Interaction Meta-Learning (PatSiML), designed to overcome these challenges. To counteract supervision collapse, we have developed a patch matching metric strategy based on the Transformer architecture to transform input images into a set of distinct patch embeddings. This approach dynamically creates task-specific embeddings, facilitated by a graph convolutional network, to formulate precise matching metrics between the support classes and the query image patches. To enhance the integration of semantic knowledge, we have also integrated a label-assisted channel semantic interaction strategy. This strategy merges word embeddings with patch-level visual features across the channel dimension, utilizing a sophisticated language model to combine semantic understanding with visual information. Our empirical findings across four diverse datasets reveal that the PatSiML method achieves a classification accuracy improvement of 0.65% to 21.15% over existing methodologies, underscoring its robustness and efficacy.

## 1. Introduction

Deep neural networks have become a dominant approach in the current image classification field. However, the high accuracy of deep learning usually relies on the large-scale labeled dataset, which can be infeasible in practical applications like medicine, military, and finance due to privacy concerns, security issues, or high labeling costs [1]. When the training dataset is small, the network is particularly prone to overfitting during training, so that the trained model has a weak generalization ability and the recognition accuracy is drastically reduced [2]. This is the challenge of few-shot learning (FSL).

Meta-learning, which has achieved notable progress in recent years [3,4,5], is one effective way of solving FSL problems. Unlike traditional machine learning algorithms, meta-learning utilizes knowledge and insights acquired from past historical tasks to guide the learning process for new tasks [6]. Metric-based meta-learning methods, such as ProtoNet [7] and Relation-Net [8], have shown promise in FSL scenarios. The flexible nature of these models reduces the need for extensive adjustments in certain few-shot classification tasks.

When using metric-based meta-learning methods in the domain of few-shot image classification, it is necessary to fully explore the inner information contained in the images and effectively utilize information from other approaches, due to an insufficient number of training samples. Unfortunately, many metric-based meta-learning approaches for few-shot image classification fail to effectively utilize local image information and additional semantic knowledge. The success of these approaches heavily relies on the quality of metric learning, which can lead to suboptimal performance if the metrics are inadequate or inaccurate. Additionally, utilizing metrics with whole-image features may overlook crucial information needed for novel tasks, resulting in supervision collapse [2].

Supervision collapse poses a significant challenge within meta-learning algorithms. This phenomenon occurs when the trained network only represents the classes present in the training set, thereby discarding potentially valuable information that is crucial for handling out-of-distribution classes. This issue arises from the network’s inclination to minimize losses during training. The reasons for supervision collapse can be attributed to two factors:

(1) Preferences for base categories. Category preferences arise from feature bias towards base classes in methods like DynamicFSL [9] and Meta-baseline [3]. Typically, these methods use fixed-weight feature extractors in the pre-training stage of supervised learning. As a result, the extracted features tend to favor base classes over new categories, resulting in a loss of discriminative ability for the novel categories.
(2) The overwhelming of local features. When performing image classification, some important targets in the image usually exist in the local range of the image, and a direct comparison with the whole-image features is not always the best. In natural images, the overwhelming of local features occurs when an image depicts multiple distinct objects or entities. Many metric-based meta-learning methods [3,7,8,9] extract features representing a single object from an entire image, ignoring other objects that may be contained in the image background. This method calculates similarity metrics between support classes and query images, potentially overlooking important local information and leading to ambiguous classification outcomes.

When using metric-based meta-learning methods in the domain of few-shot image classification, labeled images are limited, making it challenging to adequately represent a category solely based on visual features from a single image or a few samples used as metric prototypes. This limitation can hinder the model’s ability to generalize effectively in few-shot scenarios, resulting in an unreliable classifier. To address this issue, leveraging multimodal learning by incorporating textual information such as semantic knowledge can enhance the learning of concepts.

**Figure 1:**
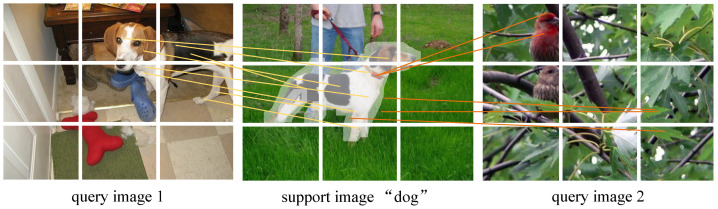
Given a support image and a query image, our approach first extracts the patch embedding of the support image (chunking and encoding the image) and enhances the feature discriminative properties with semantic knowledge (identifying key regions in the local image). Finally, the patch matching metric is used to achieve feature similarity comparison between local key information. In this approach, even if the background of query image 2 is similar to the support image, it is still not easily predicted as the label of the support image.

Our method uses a chunking strategy, adding semantic information to achieve feature similarity comparison. This paper aims to address supervised collapse and efficient use of semantic knowledge by fully utilizing local features of images and prior semantic knowledge. We propose a Patch Matching Metric-based Semantic Interaction Meta-Learning (PatSiML) method for few-shot image classification. As seen in Figure 1, this method seeks to enhance the accuracy of few-shot image classification by matching metrics between local key information. The following contributions are made in this paper:

(1) To address preferences for base categories in supervision collapse, a self-supervised learning method is introduced for pretraining the feature extractor using knowledge distillation. The target network can learn more generalized features, reducing the dependency on categories’ labels.
(2) This work proposes a new image matching metric strategy that utilizes image patch embeddings to achieve semantic similarity calculation at the patch level. Additionally, by introducing a graph convolutional network (GCN)-based method for patch embedding construction, it addresses the overwhelming of local features in supervision collapse, filling the gap in patch matching metric.
(3) We introduce a channel semantic interaction strategy to enhance the fusion of semantic knowledge and visual features in few-shot classification. By leveraging multimodal fusion, this methodology improves semantic understanding and task adaptability in meta-learning models.
(4) Our method demonstrates improved classification accuracy, ranging from 0.65% to 21.15% over established methods across four benchmark datasets.

## 2. Related Works

**Supervision collapse [2].** Previous research [2] has utilized self-supervised pretraining as an alternative to supervised training. This approach aims to learn more generalized features and address the issue of supervision collapse, which can arise from the preference for base categories. In this paper, we draw on [10] and employ a self-supervised method based on masked image modeling for class-independent training to acquire more generalizable features. The goal of this method is to provide a solution for the supervision collapse issue caused by local feature overwhelming.

In order to mitigate supervision collapse, SSFormer [11] combines the self-attention mechanism and divides each input image into multiple identical image blocks. It preserves contextual information while enabling local features to communicate their own information. Unlike earlier methods, our technique takes advantage of Transformer’s ability to process images in patches. Its self-attention mechanism allows us to facilitate more extensive interactions among nearby features. Therefore, instead of using CNN for feature extraction in this paper, we employ a Transformer-like ViT.

**Efficient utilization of semantic knowledge.** There are two key issues in the use of additional semantic knowledge. One issue is how semantic knowledge is extracted, and the other is how semantic knowledge and visual features are effectively combined. Existing approaches [12,13] often use Word2vec and GloVe to extract semantic knowledge. However, based on the experimental results of these approaches [12,13], these old methods are not very good at understanding text [12]. We will explore the latest natural language models for semantic extraction, such as BERT and CLIP, in this paper. There are essentially two categories in which semantic knowledge and visual elements are combined. One category [12,14] uses semantic vectors to assist the adjustment of visual features, playing a pivotal role in enhancing the classifier or loss function. However, this approach may oversimplify the information gap between textual and visual features, leading to potential semantic bias. Another category [15,16] relies on multimodal fusion, where semantic knowledge and visual features are interactively fused, mitigating semantic bias but risking the overuse of semantic knowledge.

SP-CLIP [16] serves as the foundation for our class-label-assisted channel semantic interaction approach. This paper draws inspiration from SP-CLIP, which integrates semantic knowledge and visual features in both spatial and channel dimensions at the underlying level. Complementary information between different modalities of semantic knowledge and visual features is captured by this multi-modal fusion. However, using underlying features for multimodal fusion can lead to the overutilization of semantic knowledge and impair classification performance. Additionally, spatial dimension fusion is not suitable for dispersed patch blocks. Therefore, the method proposed in this paper adapts the metric strategy according to patch blocks while implementing the fusion of semantic knowledge and visual features only in the channel dimension.

## 3. Methodology

### 3.1. Framework

**Figure 2:**
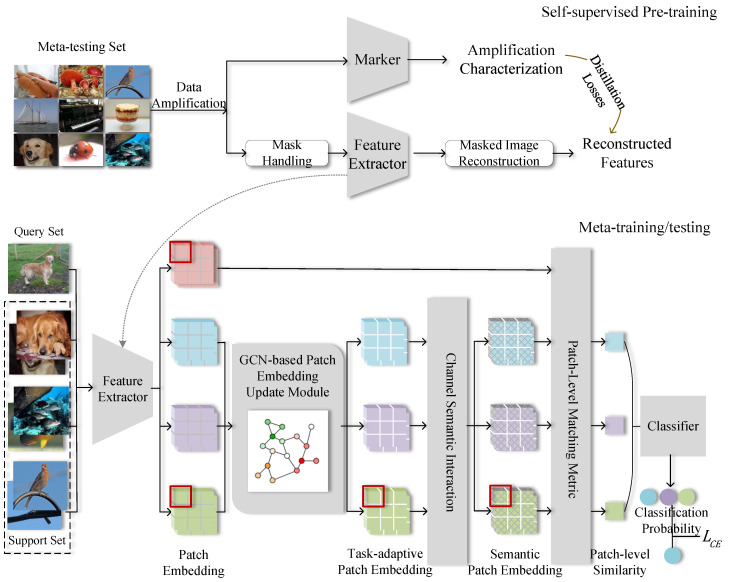
Semantic interaction meta-learning approach based on patch matching metrics. The PatSiML algorithm as a whole follows a general pretraining, meta-training, and meta-testing process.

The framework of the proposed Patch Matching metric-based Semantic interaction Meta-Learning (PatSiML) in this paper is illustrated in Figure 2. It simulates the implementation of PatSiML on a three-way two-shot few-shot image classification task, assuming that each image is partitioned into nine patch embeddings by a feature extractor.

In our methodology, we employ the self-supervision algorithm iBot [17] as a feature extractor for pretraining. Leveraging the inherent capabilities of iBot, the target network acquires generalized visual feature expression abilities through knowledge distillation with a teacher–student network framework. This step is simplified in Figure 2. There are three stages in our methodology: the pretraining stage, the meta-training stage, and the meta-testing stage.

During the pretraining stage, the PatSiML algorithm diverges from general meta-learning algorithms by employing a self-supervised learning approach that relies on masked image modeling. The entire network is divided into two pathways. One pathway employs a tagger as the teacher network to acquire augmented features, while the other pathway utilizes the student network, acting as the backbone of the feature extractor, to engage in masked image modeling. The primary objective during training is to minimize the distillation losses between the augmented features and the reconstructed features. The feature extractor weights after self-supervised pretraining are retained and transferred to the feature extractor in the meta-training stage.

In the meta-training stage, the support set images are encoded to patch embeddings for each class by the feature extractor. We utilize the patch embeddings from the support set as nodes to create a semantic graph, which represents the relationships between patch embeddings. Then, the nodes are updated by GCN to obtain the task-adaptive patch embeddings, which are then input into the channel semantic interaction module. The semantic cue features of the labels are fused and interact with the visual features in the channel dimension for semantic complementation and guidance. Subsequently, the patch-level similarity between the query image and each class of the support set is calculated by the matching metrics module. Finally, a classifier is used to predict the results and calculate the categorical cross-entropy loss to update the whole network.

The meta-testing stage corresponds to the process of downstream few-shot image classification tasks. The process is similar to the meta-training stage in that there are no network weight updates and the classifier directly outputs predictions.

### 3.2. Self-Supervised Pretraining

In this study, the input image is partitioned into smaller patches to alleviate the supervision collapse problem caused by the overwhelming of local features. Particularly, the feature representation of each patch of an image typically has greater semantic significance than that of the entire image since each local region typically has only one principal target entity. However, due to the lack of labeling information for these fine-grained regions, a self-supervised methodology is required to encode and train the information of each local region. This approach aims to capture the semantic features of individual local regions within the image. The primary objective is to extract unlabeled features while addressing the supervised collapse resulting from the class preference of features.

Masked Image Modeling (MIM) [18] meets the above requirements by performing random region masking on the image and reconstructing the regional features of the original image. iBOT [17] is a self-supervised framework that models MIM as Knowledge Distillation (KD). Specifically, the online tokenizer functions as the teacher network, conducting masked prediction. The target network is designated as the student network, enabling it to self-distill knowledge from the teacher network through BERT-style pretraining to obtain a generalized visual feature representation. Additionally, since Transformer-based neural networks need to divide the image into patches first, they are well suited for this self-supervised pretraining approach in which MIM is used pre-task.

We employ iBOT [17] directly for pretraining the target networks, namely Vision-Transformer and Swin-Transformer. By leveraging the patch embedding constraints introduced through self-supervised pretraining based on MIM, the Transformer-based target network is induced to acquire an embedding space that generates semantically valuable feature representations for each patch. This measure aims to acquire the target network’s weights and share them to the backbone for use in the meta-training stage.

### 3.3. Patch Matching Metric Strategy

Conventional methods of image classification typically take class embeddings as image features and then input these features into the classification layer for prediction. However, instead of utilizing the category embeddings, the proposed patch matching metric strategy makes full use of the patch embeddings output generated by Transformer. This strategy calculates the patch-level semantic similarity between the patch embeddings of the support class and query image, aiming to achieve an image patch matching metric. As a result, supervised collapse problems caused by the overwhelming of local features are avoided.

#### 3.3.1. GCN-Based Patch Embedding Construction

Given the query image *q* and the support set $S={\left\{S^{c}\right\}}_{c=1}^{N}$ in the N-way K-shot scenario, the support and query images are first transferred to a feature extractor (Transformer series network). Each image is evenly partitioned into *U* patches, and the outputs are the feature descriptors of each patch, namely patch embedding $X^{\mathrm{patch}}$, the output contains the following: (1) patch embeddings of a query image $X_{q}^{\mathrm{patch}}\in R^{U\times d_{v}}$, (2) patch embeddings for a support set image $X_{s}^{\mathrm{patch}}\in R^{U\times d_{v}}$, and $d_{v}$ is the channel dimension of output image features.

**Figure 3:**
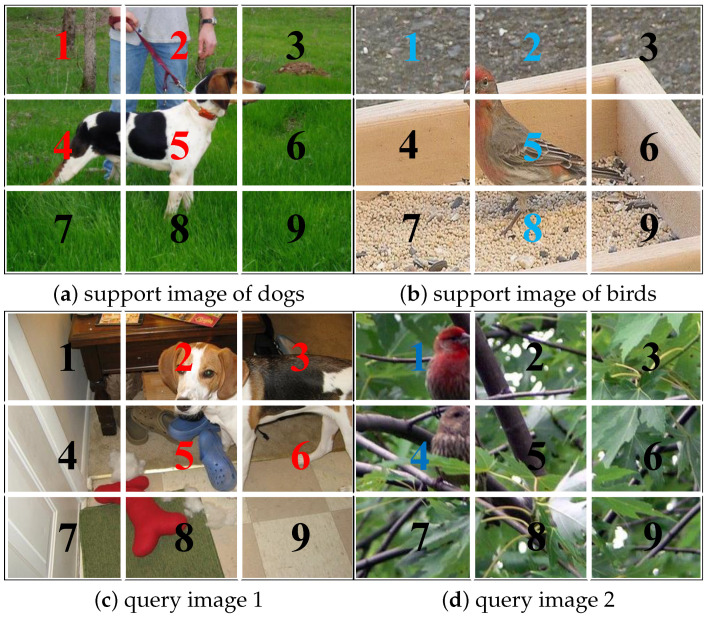
Background misdirection for patch embedding matching. As shown in (**a**), the main target “dog” occupies patches 2, 4, and 5, and the background “grass” occupies patches 1, 3, 6, 7, 8, and 9. (**d**) shows that the query image 2, with the background of “leaves” occupying most of the space, is more likely to be predicted as similar to the support image (**a**). Similarly, shown in (**b**,**c**), the background color of query image 1 closely resembles that of the support image of birds. Therefore, there is a possibility of misjudgment due to the misguidance of the background similarity.

**Background misdirection.** Using patch-level features directly for similarity computation may lead to misguidance due to the influence of background blocks [19,20]. An example of background misguidance is shown in Figure 3.

**Patch embedding semantic graph.** The issue of background misguidance arises because directly using patch embeddings for similarity calculations ignores the contextual semantic relationships among background blocks. GCN [21] can reduce such contextual misguidance by propagating features through the graph and strengthening the semantic links between patch embeddings. The key idea of GCN is to use the adjacency matrix of the graph to infer the relationships between nodes and to propagate the features through convolutional operations on the graph structure. Each node in the graph is influenced by adjacent nodes and more distantly linked nodes, continually updating its state until the final equilibrium. The closer the relationship, the greater the influence of linked nodes. The specific implementation for construction and updating of the patch embedding semantic graph is described next.

First, assuming that an image is characterized by *U* patch embeddings, a patch embedding of a support image is considered as a node of the patch embedding semantic graph. As a task comprises N×K support images, there is a set of graph nodes $S=\{s_{i}|i=1,2,\ldots ,NKU\}$.

Second, the set of edges of the graph is defined as $E=\{e_{ij}|i=1,2,\ldots ,NKU;j=1,2,\ldots ,NKU\}$. The edge value $e_{ij}$ characterizes the semantic similarity between two patch embeddings, and the semantic similarity is calculated by cosine similarity. If the two patch embeddings are from the same class, only the semantic similarity will be calculated. Otherwise, the edge value is directly set to 0. The specific formula is as follows:

(1) $$e_{ij}=1\left[c_{s_{i}}=c_{s_{j}}\right]\cos\left(X_{s_{i}}^{\mathrm{patch}},X_{s_{j}}^{\mathrm{patch}}\right),$$

(2) $$\cos\left(X_{s_{i}}^{\mathrm{patch}},X_{s_{j}}^{\mathrm{patch}}\right)=\frac{X_{s_{i}}^{\mathrm{patch}}{\left(X_{s_{j}}^{\mathrm{patch}}\right)}^{T}}{∥X_{s_{i}}^{\mathrm{patch}}∥∥X_{s_{j}}^{\mathrm{patch}}∥},$$

where $c_{s_{i}}$, $c_{s_{j}}$ represent the class of node $s_{i}$ and $s_{j}$ respectively and 1[•] is true/false indicator function.

Next, we use the set of graph edges E to generate the adjacency matrix $A={\left\{a_{ij}\right\}}_{i,j=1}^{NKU}\in R^{NKU\times NKU}$. The range of edge values constructed by Equation (1) is [−1, 1]. To ensure the convergence of the network and avoid training errors resulting from negative values, the range of edge values needs to be adjusted to [0, 2]. Then, the value of each element in the adjacency matrix A is obtained as follows:

(3) $$a_{ij}=e_{ij}+1.$$

To ensure the stability of the numerical range, the adjacency matrix must be normalized. Normalization guarantees that the node degree (i.e., the number of neighboring nodes) does not introduce numerical bias in the feature propagation process and eliminates the influence of the node degree on the feature propagation. The final normalized adjacency matrix $\hat{A}$ is shown as follows:

(4) $${\hat{A}}_{\mathrm{random}}=D^{-1}\left(A+I\right)$$

(5) $${\hat{A}}_{\mathrm{symmetry}}=D^{-1/2}\left(A+I\right)D^{-1/2},$$

where $D={\left\{d_{ij}\right\}}_{i,j=1}^{NKU}\in R^{NKU\times NKU}$ denotes the degree matrix of adjacency matrix. D is a diagonal matrix. Each node’s degree is indicated by the degree matrix’s diagonal elements, i.e., $d_{ii}=\sum_{j}a_{ij}$. The introduction of the degree matrix helps to mitigate issues related to self-transmission. Equation (4) represents random normalization, while Equation (5) corresponds to symmetric normalization. These are two common normalization methods employed to maintain training stability. The experiments in Section 4.2 will further compare the applicability of the proposed method to different normalization methods.

Before updating the semantic patch embedding graph, the patch embeddings in the support set must be pre-mapped to every graph node in advance. We integrate the patch embeddings of the support images $\left\{X_{s_{i}}^{\mathrm{patch}}|i=1,2,\ldots ,NKU\right\}$ into initial features of nodes. The patch embeddings of all the support images are concatenated together into a support matrix $X_{s}^{\mathrm{patch}}\in R^{NKU\times d_{\nu }}$. Each row of the matrix corresponds to the initial features of each node of the graph, constituting an initial feature matrix $\Psi^{0}=X_{s}^{\mathrm{patch}}$ of the graph.

**Figure 4:**
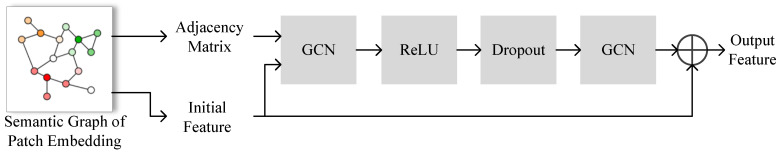
GCN-based patch embedding update module.

The core of task-adaptive patch embeddings using GCN lies in updating of semantic patch embedding graph. During updates, only the feature information associated with the graph nodes is modified, while the graph edges remain unchanged. This approach aims to make the patch embeddings task-specific based on the semantic similarity between patch embeddings and maintain the stability of the graph edge information, i.e., the initial semantic similarity. In this paper, we mainly rely on the final graph node features for subsequent processing while keeping the edge representation information in the graph unchanged. Figure 4 illustrates the GCN-based patch embedding update module.

The patch embeddings are propagated in the graph based on the following equation:

(6) $$\Psi^{b+1}=\sigma \left(\hat{A}\Psi^{b}W\right),b=\left\{0,\ldots ,B-1\right\}.$$

where $\hat{A}$ denotes normalized adjacency matrix, B stands for the number of steps to update steps, B=2, W represents learnable matrix, and σ(•) represents the ReLU function.

Finally, after propagation of the patch embedding matrix B times, the final task-adaptive patch embedding matrix ${X^{\prime }}_{S}^{\mathrm{patch}}$ is obtained.

#### 3.3.2. Patch Matching Metric

This section introduces a matching metric on patch embeddings to replace the meta-learning approach that uses overall image features in order to fully utilize local features of the image. DN4 [22] takes the vector corresponding to the pixel block in the feature map as a local descriptor and obtains similarity through several local descriptors in the support class that are closest to the query image. Based on this distance measurement, we propose a patch matching metric strategy for patch embeddings.

Given the patch embedding matrix $X_{q}^{patch}$ of a query image q and the task-adaptive patch embedding matrix ${X^{\prime }}_{S^{c}}^{patch}$ of the support class *c*, the patch-level similarity matrix of the query image concerning the class *c* can be obtained as $\;H^{c}\epsilon R^{U\times KU}$:

(7) $$H^{c}\left(X_{q}^{patch},{X^{\prime }}_{S^{c}}^{patch}\right)=\frac{X_{q}^{patch}{{(X}^{\prime }}_{S^{c}}^{patch}{)}^{T}}{∥X_{q}^{patch}∥∥{X^{\prime }}_{S^{c}}^{patch}∥}.$$

The distance between the *m*-th patch embedding of the query image and the *n*-th patch embedding of the support set category *c* is represented by the elements of the *m*-th row and *n*-th column of the matrix, which are denoted as $h_{m,n}^{c}$. A row in the patch-level similarity matrix $H^{c}$ represents the semantic similarity between the corresponding patch embedding of the query image and all the patch embeddings of the support class.

For the *m-th* patch embedding of the query image *q*, we find *L* patch embeddings that are most similar to it from the *m*-th row of the support matrix ${X^{\prime }}_{S^{c}}^{patch}$. Then, we sum the similarity values between these *L* patch embeddings of the support class *c* and the *m*-th query patch embedding. The similarity of the *m*-th patch embedding of the query image *q* and the support class *c* is denoted as $\underset{n\in 1,\ldots ,KU}{Top\_L}\left(H_{m,n}^{c}\right)$. In this paper, *L* is set as the hyperparameter of the patch matching metric. Finally, we calculate the patch-level similarity ${PM}^{c}$ between the query image *q* and the support class *c* as follows:

(8) $${PM}^{c}=\sum_{m=1}^{U}\underset{n\in \{1,\ldots ,KU\}}{{\mathrm{Top}}_{L}}\left(H_{m,n}^{c}\right).$$

The patch level similarity ${PM}^{c}$ denotes the similarity between the query image and the support class. The higher the similarity, the greater the probability that the query image will be predicted to that support class.

**Figure 5:**
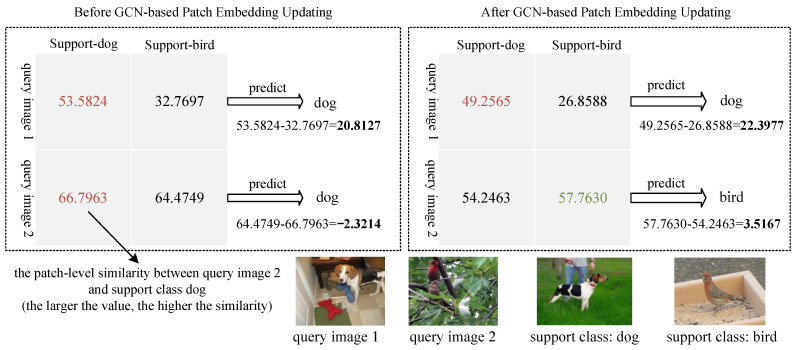
Change in patch-level similarity between the query image and support class before and after GCN-based patch embedding updating. For query image 2, the prediction is correct after updating, which corrects the previous wrong prediction.

Figure 5 shows the change in the patch-level similarity between query images and the support classes before and after the GCN-based patch embedding updating(backbone: Vit-Small, *L* = 1). As can be seen from Figure 5, the similarity difference increases for the two query images after updating the patch embeddings based on GCN, making it easier to distinguish between categories.

### 3.4. Label-Assisted Channel Semantic Interaction Strategy

In few-shot vision tasks, the use of extra semantic information is crucial to performance. In this study, we not only extract high-level semantic characteristics related to class labels using CLIP as a semantic extractor but also propose a channel semantic interaction strategy to optimize the interaction between semantic knowledge and visual features through multimodal fusion.

**Figure 6:**
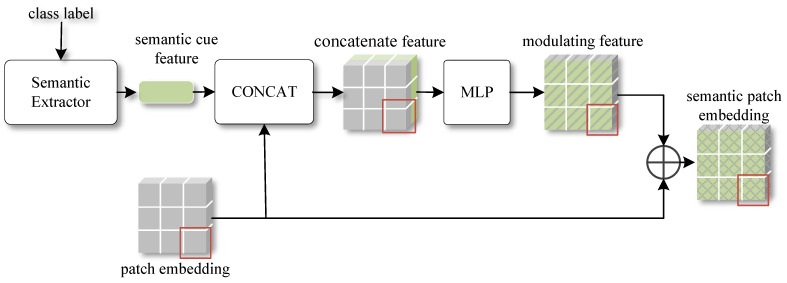
The structure of the channel semantic interaction module. The small red squares in the figure represent a patch embedding that compose the complete features of the image. In this paper, we utilize the channel semantic interaction module to implement a label-assisted channel semantic interaction strategy.

Multimodal interaction can mine the complementary information between modalities such as image and text to obtain more comprehensive features and provide a more accurate semantic understanding. The simplest multimodal semantic interaction method fuse features from different modalities through vector concatenation, vector weighted summation, or other approaches. However, these approaches lack sufficient interaction between modalities, and the connection between them is relatively weak. Semantic interaction can be conducted in the channel dimension or the spatial dimension. Compared to the spatial dimension, semantic interaction in the channel dimension exhibits better robustness to local changes. Channel information usually represents global features of an image (e.g., color, brightness, etc.), making it spatially insensitive. When small transformations or distortions occur in the image, the fusion in the channel dimension generally maintains model performance in a relatively stable manner. The channel semantic interaction module is depicted in Figure 6.

The structure of channel semantic interaction module is shown in Figure 6, where the small red squares represent one of the patch embeddings that make up the complete features of the image. First, the class labels are passed through the semantic extractor to generate semantic cue feature vectors. Next, the semantic cue feature is concatenated with all patch embeddings along the channel dimension. Then, the concatenated features are input into the MLP module, which performs channel dimension modulation and semantic enhancement of the visual features. Finally, the modulating features are added to the original patch embedding to obtain the final semantic patch embedding. Through the channel semantic interaction mechanism assisted by class labels, semantic knowledge can be integrated and interacted with visual features. The visual features are tuned channel-by-channel using the text information of class labels to improve the discriminative properties of features and promote the task adaptability of the meta-learning model. The specific implementation steps are described in detail next.

First, CLIP [23] is utilized as a semantic extractor and the input label is expanded with a text template, which is “A photo of a [class name]”. For example, for the label “cat”, the text of the expanded class label is “A photo of a cat”. We feed the label $y_{c}$ into CLIP and then obtain the semantic cue feature vector $g_{c}$ for category *c*:

(9) $$g_{c}=g\left(y_{c}\right)\in R^{d_{g}},$$

where $d_{g}$ denotes the dimension of the semantic cue feature.

Secondly, each patch embedding of an image with support class c is concatenated with semantic cue feature in the form $\left[{X\prime }_{s_{i}^{c}}^{patch},g_{c}\right]$. This concatenated representation is transferred to a two-layer MLP for channel interaction to obtain the modulated feature. The i-th (i∈[1,KU]) modulated vector of support class c, $\gamma_{i}^{c}\;$, is computed as follows:

(10) $$\gamma_{i}^{c}=\sigma_{2}\left(W_{2}\;\sigma_{1}\left(W_{1}\left[X_{s_{i}^{c}}^{\mathrm{patch}\;\prime },\;g_{c}\right]+b_{1}\right)+b_{2}\right),$$

where $W_{1}$ and $W_{2}$ denote the weight parameters of the first and second linear layers of MLP, respectively; $b_{1}$ and $b_{2}$ denote the bias of the first and second linear layers of the MLP; $\sigma_{1}\left(•\right)$ and $\sigma_{2}\left(•\right)$ are sigmoid activation functions.

Finally, the modulation vector is added to the corresponding patch embedding to adjust the visual features of each channel, yielding the final semantic patch embedding as follows:

(11) $$z_{i}^{c}={X\prime }_{s_{i}^{c}}^{patch}+\gamma_{i}^{c}.$$

The matrix of the semantic patch embedding for support class c is ${Z\prime }_{S^{c}}^{patch}\in R^{KU\times d_{v}}$, where each row corresponds to a semantic patch embedding $\;z_{i}^{c}$. In this paper, the channel semantic interaction is carried out after the task-adaptive patch embedding is obtained in Section 3.3.1. The semantic patch embedding matrix $\;{Z\prime }_{S^{c}}^{patch}$ replaces ${X^{\prime }}_{S^{c}}^{patch}$ in Equation (7) in Section 3.3.2. Then, the patch-level similarity is calculated according to Equation (8) in order to realize the patch-level matching metric.

## 4. Experiments

In this section, we evaluate the performance of PatSiML algorithm on four few-shot image classification datasets. First, we describe the specific implementation details of the experiment. The experimental setting for the PatSiML algorithm is listed in Table 1. Second, a comparison is conducted with other widely used methods. Subsequently, the effects of the adjacency matrix normalization method, hyperparameters in the matching metric module, and the semantic extractor on the performance of the algorithm are explored. Finally, ablation experiments are performed to verify the patch matching metric strategy and the label-assisted channel semantic interaction.

### 4.1. Implementation Details

**Datasets and Backbone.** Like the classical methods [9,10,11,16] in the field of few-shot image classification, four public few-shot image classification benchmark datasets are used in experiments: Mini-ImageNet [24], Tiered-ImageNet [25], CIFAR-FS [26], and FC-100 [27]. To balance model complexity and efficiency, our algorithm PatSiML uses the relatively small Transformer architectures, Vit-Small [28] and Swin-Tiny [29].

**Training details.** We divide the training process into two stages: self-supervised pretraining and meta-training. Note that no extra data are used for pretraining. The architecture is exclusively trained on the corresponding meta-training set using iBOT [17]. The Vit-Small and Swin-Tiny are trained with a batch size of 512 for 1600 epochs (with Tiered-ImageNet set to 800) and 800 epochs. The meta-training stage has 30 epochs, each with 200 episodes. We evaluate 600 “*N*-way *K*-shot” tasks to select the best set of parameters as the final meta-training model parameters.

**Test details.** During the testing stage, 1000 “N-way K-shot Q-query” tasks are randomly selected from the meta-test set (Q=15). We use the average classification accuracy of the 1000 tasks as the quantitative evaluation metric and report a 95% confidence interval.

**Parameters.** The self-supervised pretraining follows the previous work [17]. The parameters of our meta-learning network include parameters of feature extractor, task adaptive patch embedding update, and channel semantic interaction module. During meta-training, we use AdamW [30] with a weight decay of $5\times 10^{-2}$. Channel semantic interaction parameters are updated with an initial learning rate of $2\times 10^{-4}$, and the initial learning rates of the other two parts are updated with $10^{-6}$. The learning rate is adjusted using the cosine annealing algorithm [17], with a cosine annealing period of 600.

### 4.2. Experiments of Different Adjacency Matrix

In the process of performing patch embedding updates based on GCN, the adjacency matrix of the patch embedding semantic graph needs to be constructed, and the adjacency matrix needs to be reasonably normalized. Experiments are conducted on Mini-ImageNet and Tiered-ImageNet to determine the form of the adjacency matrix. The experimental results are shown in Table 2 and Table 3. We conduct the experiments by removing the semantic interaction strategy of the channels (i.e., Experiment (B) in Section 4.5). If the two patch embeddings are of the same category, the simple adjacency matrix assigns an edge value of 1, otherwise, an edge value of 0 is assigned. Each element of the adjacency matrix *A* is $a_{ij}=1\left[c_{s_{i}}=c_{s_{j}}\right]$. “Our adjacency matrix” has been described in Section 3.3.1.

The experiments in Table 2 and Table 3 indicate that our adjacency matrix constructed with symmetric normalization performs better when the backbone is Vit-Small. It is constructed with stochastic normalization and outperforms the other combination forms when Swin-Tiny is the backbone. The two networks’ different normalizations may be related to structural differences. Vit-Small uses a global attention mechanism, and symmetric normalization can maintain the symmetry of the adjacency matrix, which helps to retain global information in the graph. In contrast, Swin-Tiny, a sliding-window-based Transformer model, pays more attention to the local information, and the introduction of stochastic normalization can better adapt to locally focused networks.

In addition, these results verify that the neighbor matrix constructed using the semantic similarity between patch embeddings has performance enhancement. This is because it focuses more on capturing fine-grained semantic information, which helps the model better understand the similarities and differences between patch blocks and improves the graph representation.

The normalization of the adjacency matrices in our method is as follows unless otherwise noted: symmetry for Vit-Small and random for Swin-Tiny.

### 4.3. Selecting Hyperparameters of Patch-Level Matching Metric

**Figure 7:**
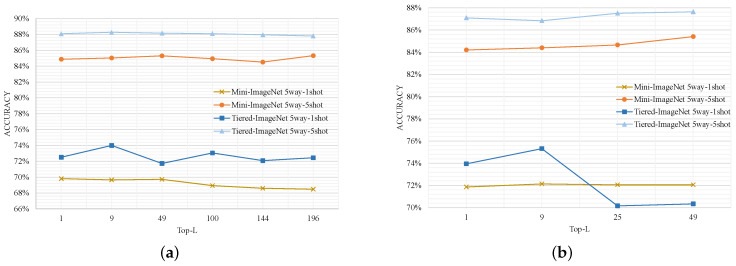
The effect of the patch-level matching metric’s hyperparameter L. (**a**) Vit-Small; (**b**) Swin-Tiny.

The hyperparameter *L* in our patch matching metric plays a crucial role. It determines how many of the most similar patch embeddings from the support set are used to calculate the similarity with a patch embedding of the query image. On mini-ImageNet and tiered-ImageNet datasets, we test the effect of *L* on the classification accuracy of FSL, as shown in Figure 7.

We conduct experiments by using the method that removes the semantic interaction strategy in this paper. Since the input image size is 224 × 224 for mini-ImageNet and tiered-ImageNet, the number of patch embeddings output from Vit-Small is 196, and that output from Swin-Tiny is 49. The experimentally chosen *L* is the square of an integer.

From Figure 7a, it is evident that employing a smaller *L* for one-shot tasks has more favorable outcomes when Vit-Small is utilized as the backbone. The highest accuracy is achieved with *L* values of 1 and 9 on Mini-ImageNet and Tiered-ImageNet datasets, respectively. For a five-shot task, varying values of *L* do not significantly impact experimental results, with peak accuracy observed at *L* = 196 (Mini-ImageNet) and *L* = 9 (Tiered-ImageNet).

Figure 7b shows that a smaller *L* also leads to improved accuracy for one-shot tasks when Swin-Tiny serves as the backbone. Particularly on Tiered-ImageNet, optimal performance is attained at *L* = 9 while performance substantially deteriorates at *L* = 25 or 49. For both datasets and the five-shot task, larger values of *L* correspond to higher accuracies, with peak performance achieved at *L* = 49 (Mini-ImageNet and Tiered-ImageNet).

Combining the above experiments, the setting of the hyperparameter L in this thesis follows Table 4 unless otherwise stated. The values of *L* on the Mini-ImageNet and Tiered-ImageNet datasets follow the conclusions of the above experiments. The input image size of CIFAR-FS and FC100 datasets is 84 × 84. Therefore, for ViT-Small, the maximum values of *L* are 25 (one shot) and 125 (five shot), and the smaller value of *L* is chosen for the one-shot task. For the five-shot task, the number of patches that can form a complete image is chosen as 25. The maximum value of *L* on the Swin-Tiny backbone is not affected by the size of the input image, so we choose the same value of *L* as the Mini-ImageNet and Tiered-ImageNet datasets.

### 4.4. Few-Shot Image Classification Experiments

Experiments are conducted to compare our method PatSiML with the advanced and classical methods in the field of few-shot image classification. The scenarios are set to five-way one-shot (5W1S) and five-way five-shot (5W5S) tasks, which means the method has to identify images from five novel classes based on one or five labeled images provided per category.

Experiments are conducted on Mini-ImageNet and Tiered-ImageNet datasets to compare our method, PatSiML, with several popular methods listed in Table 5. The optimal algorithm is represented by bold values, while the suboptimal method is indicated with an underline. It is evident that PatSiML performs at its best. PatSiML-Swin with Swin-Tiny outperforms the state-of-the-art algorithms by 1.53% (Tiered-ImageNet) and 2.02% (Mini-ImageNet) on the one-shot challenge. Although PatSiML is 0.05% less accurate than MetaQDA [31] in the five-shot task, the number of parameters is smaller. Therefore, its overall performance is better. Additionally, PatSiML outperforms the other four approaches that use a Transformer as the backbone with a 0.96% to 8.88% advantage.

The experimental results of the algorithm on the FC100 and CIFAR-FS datasets are presented in Table 6. Our method, PatSiML, demonstrates the best performance on the CIFAR-FS dataset. It also shows its advancement on the FC100 dataset, which is more difficult in few-shot learning (FSL). On the one-shot task, PatSiML-ViT outperforms SP-CLIP by 2.08%, which uses the same semantic extractor CLIP as ours. It indicates that our approach achieves better results by using patch embedding to interact with channels of semantic information. On the five-shot task, using the same self-supervised pretraining model, the classification accuracy of PatSiML-Swin outperforms that of FewTURE [10] by 1.22%.

In summary, our PatSiML shows the most competitive performance available on all four popular few-shot image categorization datasets. In particular, PatSiML-Swin outperforms PatSiML-ViT on almost all of these datasets.

### 4.5. Ablation Experiments

In order to validate the effectiveness of each strategy used in our PatSiML, we conduct ablation experiments on mini-ImageNet and tiered-ImageNet datasets. The settings are demonstrated in Table 7. The results are shown in Table 8 and Table 9.

Experiment (A) removes the patch matching metric and the channel semantic interaction strategy, uses the global level metric approach of ProtoNet [7], and retains the self-supervised pretraining approach.

In Experiment (B), we omit the channel semantic interaction strategy. When (B) and (A) are compared, it can be shown that (B) improves performance by more than 2.50%. This can be attributed to the patch matching metric strategy’s intelligent use of local features. The graph convolutional network strengthens the link between the local features, and the patch matching metric implements a similarity measure between local features.

In Experiment (C), the patch matching metric strategy is removed. Compared to experiment (A), the results indicate that experiment (C) is more accurate using channel semantic interactions, even though experiment (C) was conducted on a class prototype representing global features. The inclusion of semantic knowledge in the channel dimension improves the discriminative nature of the visual features and facilitates the task adaptation of the meta-learning model, which in turn improves the performance of few-shot image classification.

Experiment (D) is the replacement of self-supervised pretraining with supervised pretraining. As can be seen from Table 4 and Table 5, Experiment (D) is even less effective than (A). It demonstrates that our method’s prerequisites and foundations are self-supervised pretraining. The self-supervised training complements the Transformer structure and combines with the matching metric strategy in this section to implement the meta-learning idea of patch matching metrics. Thus, this approach effectively prevents the supervised collapse problem and improves the generalization of the meta-learning model.

Experiment (E) is PatSiML, as proposed in this paper. Comparison of Experiments B, C, D, and E reveals that each of the constituent strategies of PatSiML contributes positively to the algorithm’s performance on the few-shot image categorization task. The strategies can be ranked, from highest to lowest, as follows: self-supervised pretraining, patch matching metric, and semantic interaction. Self-supervised pretraining has the most obvious benefit to the algorithm because it explores richer information beyond the class label. Moreover, self-supervised learning encourages the model to understand the context of the image, which helps the patch matching metric strategy. The patch matching metric strategy also has a relatively large beneficial effect on the algorithm because it replaces the metric of using the whole image feature for comparison and avoids the lack of utilization of local features. Semantic interactions have a relatively small impact on the performance, as semantic knowledge mainly serves to complement the visual features.

### 4.6. Selecting Helpful Semantic Extractors

In this section, the PatSiML algorithm attempts to utilize semantic knowledge to tune the visual features channel-by-channel to improve the discriminative properties of the features. Consequently, the robustness of the semantic knowledge is very important, and it is necessary to explore the effect of the semantic extractor on the performance of the algorithm. The experiments compare the performance of the PatSiML algorithm on Mini-ImageNet and Tiered-ImageNet datasets when three models, CLIP [23], SBERT [36] and GloVe [37], are used as semantic extractors, and the experimental results are presented in Table 10.

The three types of semantic extractors have all been pretrained on a large-scale corpus and can be directly utilized. In the experiments, the dimension of the class label semantic vectors extracted using CLIP is 512, the dimension of the semantic vectors extracted using SBERT is 768, and the dimension of the GloVe extraction is 300. The experimental results in Table 9 show the following:

(1) The CLIP model works best as a semantic extractor. The CLIP model outperforms the other two semantic extractors on both Mini-ImageNet and Tiered-ImageNet datasets. The possible reason is that CLIP extracts richer and more advanced semantic features from class names. In contrast, SBERT and GloVe can only accept class labels as input to their encoders, resulting in the output of word vectors representing the class names (if there are multiple words in the class labels, the output word vectors are averaged). Given that CLIP is trained to align visual and semantic spaces, its semantic extractor is accurately a more customizable semantic knowledge, e.g., in this section, “A photo of a class name” is used as a text template for customization of semantic cues.
(2) The semantic knowledge extracted by the algorithm in this section using the CLIP model is more robust. Compared to the first row of the table without channel semantic interaction, the approach using the CLIP model has performance improvement in both datasets, both backbone networks, and both one-shot and five-shot scenarios. On the contrary, for the other two semantic extractors, the GloVe method has lower accuracy on the five-shot task than the method without semantic extractor, and the SBERT method also suffers from performance degradation.

## 5. Conclusions

Our results demonstrate that PatSiML effectively integrates self-supervised pretraining, a patch matching metric strategy, and a class-label-assisted channel semantic interaction strategy, achieving superior accuracy. Self-supervised learning is used to pretrain feature extractors with higher generalizability. To address the issue of supervision collapse caused by the drowning of local features, images are first encoded into multiple patch embeddings using Transformer. A semantic graph is constructed to update these embeddings and measure their similarity to achieve image classification. Subsequently, the label-assisted channel semantic interaction strategy further exploits semantic knowledge to complement the visual features. We adopt a more robust semantic extractor and design a channel-based semantic interaction strategy tailored to patch embeddings, which guides visual features towards improved discriminability. Experimental results on four datasets, Mini-ImageNet, Tiered-ImageNet, CIFAR-FS, and FC100, show that our proposed PatSiML outperforms current popular methods by 0.65% to 21.15%. Ablation experiments validate the effectiveness of each strategy employed in this paper.

The PatSiML method proposed in this paper enhances the generalization of the meta-learning model, improves the performance of small sample image classification, and outperforms the existing popular methods on multiple datasets. However, the methods proposed in this paper still have some limitations and there is room for improvement as follows:

(1) The PatSiML method proposed in this paper uses class labels as semantic knowledge, which can be combined with other methods in the field of natural language processing to further optimize the semantic knowledge using category-based attribute information or textual cues to improve the robustness of the semantic knowledge source and to make full use of additional semantic cues to complement the visual features.
(2) In this paper, the PatSiML approach is dominated by Transformer networks, and large-scale Transformer networks have huge requirements in terms of computational resources and storage, making them difficult to deploy in resource-constrained environments. In practice, for hardware implementation, the model needs to be lightweight, and the number of model parameters is reduced through quantization, pruning, knowledge distillation, etc., a process that will reduce the accuracy of the method to a certain extent. In subsequent practical deployment, it is necessary to reasonably choose the type of Transformer network and model lightweighting method through experiments to balance the accuracy and resource cost.
(3) In this paper, experiments are conducted only on four classical datasets of few-shot learning, and subsequent tests are prepared on other few-shot learning datasets and not few-shot learning datasets to validate the effectiveness of the method proposed in this paper.

## Figures and Tables

**Table 1:** Experimental hardware and software environment.

Environment	Parameters
Operating System	Windows 10 Enterprise 64-bit
CPU	Intel Core i9 12900K
Memory	DDR4 64 GB
GPU	Nvidia RTX 3090
Python	3.7
Cuda	11.1
Pytorch	1.7.1

**Table 2:** Results of the adjacency matrix and its normalization on Mini-ImageNet dataset (L = 1).

Type	Normalization	Vit-Small		Swin-Tiny	
		5W1S	5W5S	5W1S	5W5S
simple adjacency matrix	random	68.55 ± 0.64	83.07 ± 0.43	71.17 ± 0.62	84.03 ± 0.43
	symmetry	68.68 ± 0.64	83.76 ± 0.41	71.17 ± 0.62	84.01 ± 0.42
our adjacency matrix	random	69.56 ± 0.65	84.01 ± 0.40	**71.86 ± 0.62**	**84.20 ± 0.42**
	symmetry	**69.82 ± 0.65**	**84.88 ± 0.40**	71.50 ± 0.62	83.97 ± 0.44

Note: The values in the 5W1S and 5W5S columns of the table are accuracy rates and 95% confidence intervals, with values in percent.

**Table 3:** Results of the adjacency matrix and its normalization on tiered-ImageNet dataset (L = 1).

Type	Normalization	Vit-Small		Swin-Tiny	
		5W1S	5W5S	5W1S	5W5S
simple adjacency matrix	random	71.10 ± 0.61	85.12 ± 0.51	70.15 ± 0.92	86.03 ± 0.51
	symmetry	71.29 ± 0.60	85.67 ± 0.50	70.37 ± 0.72	86.07 ± 0.50
our adjacency matrix	random	72.37 ± 0.70	87.69 ± 0.44	**73.95 ± 0.72**	**87.09 ± 0.50**
	symmetry	**72.51 ± 0.71**	**88.08 ± 0.44**	71.09 ± 0.72	86.64 ± 0.49

Note: The values in the 5W1S and 5W5S columns of the table are accuracy rates and 95% confidence intervals, with values as percentages.

**Table 4:** Setting of the hyperparameter L in PatSiML.

Dataset	Vit-Small		Swin-Tiny	
	5W1S	5W5S	5W1S	5W5S
Mini-ImageNet	1	196	9	49
Tiered-ImageNet	9	9	9	49
CIFAR-FS	1	25	9	49
FC100	1	25	9	49

**Table 5:** Average classification accuracy on Mini-ImageNet and Tiered-ImageNet.

Method	Backbone	≈Params	Mini-ImageNet		Tiered-ImageNet	
			5W1S	5W5S	5W1S	5W5S
MAML [6]	ResNet-12	12.5 M	58.60 ± 0.61	69.54 ± 0.56	59.82 ± 0.56	73.17 ± 0.56
DynamicFSL [9]	ResNet-12	12.5 M	62.81 ± 0.27	78.97 ± 0.18	68.35 ± 0.31	83.52 ± 0.21
DeepEMD-Bert [14]	ResNet-12	12.5 M	67.03 ± 0.791	83.68 ± 0.6	73.76 ± 0.72	87.51 ± 0.75
SSFormers [11]	ResNet-12	12.5 M	67.25 ± 0.24	82.75 ± 0.20	72.52 ± 0.25	86.61 ± 0.18
LPE-Glove [32]	ResNet-12	12.5 M	68.28 ± 0.43	78.88 ± 0.33	72.03 ± 0.49	83.76 ± 0.37
SIB [33]	WRN-28-10	36.5 M	70.00 ± 0.60	79.20 ± 0.40	70.01 ± 0.54	84.13 ± 0.54
Align [34]	WRN-28-10	36.5 M	65.92 ± 0.60	82.85 ± 0.55	74.40 ± 0.68	86.61 ± 0.59
MetaQDA [31]	WRN-28-10	36.5 M	67.83 ± 0.64	84.28 ± 0.69	74.33 ± 0.65	**89.56 ± 0.79**
ProtoNet-Swin	Swin-Tiny	29.0 M	67.28 ± 0.67	82.56 ± 0.44	70.68 ± 0.71	85.81 ± 0.47
SUN [35]	Visformer-S	12.4 M	67.80 ± 0.45	83.25 ± 0.30	72.99 ± 0.50	86.74 ± 0.33
SP-CLIP [16]	Visformer-T	10.0 M	72.31 ± 0.40	83.42 ± 0.30	78.03 ± 0.46	88.55 ± 0.32
FewTURE [10]	Swin-Tiny	29.0 M	70.48 ± 0.62	84.41 ± 0.41	76.32 ± 0.87	88.70+0.44
PatSiML-ViT (ours)	Vit-Small	22.0 M	72.26 ± 0.57	85.39 ± 0.43	74.74 ± 0.69	88.90 ± 0.48
PatSiML-Swin (ours)	Swin-Tiny	29.0 M	**74.33 ± 0.56**	**85.89 ± 0.42**	**79.56 ± 0.66**	89.51 ± 0.46

Note: The values in the 5W1S and 5W5S columns of the table are accuracy rates and 95% confidence intervals, with values as percentages.

**Table 6:** Average Classification Accuracy on CIFAR-FS and FC100.

Method	Backbone	≈Params	CIFAR-FS		FC100	
			5W1S	5W5S	5W1S	5W5S
DynamicFSL [9]	ResNet-12	12.5M	61.68 ± 0.26	78.97 ± 0.18	40.81 ± 0.56	56.64 ± 0.58
SSFormers [11]	ResNet-12	12.5M	74.50 ± 0.21	86.61 ± 0.23	43.72 ± 0.21	58.92 ± 0.61
SIB [33]	WRN-28-10	36.5M	80.00 ± 0.60	85.30 ± 0.40		
MetaQDA [31]	WRN-28-10	36.5M	75.83 ± 0.88	88.79 ± 0.70		
ProtoNet-Swin	Swin-Tiny	29.0M	71.24 ± 0.45	82.47 ± 0.43	42.13 ± 0.67	57.11 ± 0.62
SUN [35]	Visformer-S	12.4M	78.37 ± 0.46	88.84 ± 0.32		
SP-CLIP [16]	Visformer-T	10.0M	82.18 ± 0.40	88.24 ± 0.32	48.53 ± 0.38	61.55 ± 0.41
FewTURE [10]	Swin-Tiny	29.0M	77.76 ± 0.81	88.90 ± 0.59	47.68 ± 0.78	63.81 ± 0.75
PatSiML-ViT	Vit-Small	22.0M	82.83 ± 0.61	90.48 ± 0.44	50.61 ± 0.59	64.09 ± 0.62
PatSiML-Swin	Swin-Tiny	29.0M	81.72 ± 0.59	90.72 ± 0.38	50.42 ± 0.58	65.03 ± 0.57

Note: The values in the 5W1S and 5W5S columns of the table are accuracy rates and 95% confidence intervals, with values as percentages.

**Table 7:** Description of ablation experiment setup.

No.	Self-Supervised Pretraining	Patch Matching Metric	Channel Semantic Interaction	Instructions
(A)	✓	-	-	Removal of patch matching metric and channel semantic interaction strategy using ProtoNet.
(B)	✓	✓	-	Removing channel semantic interaction.
(C)	✓	-	✓	Removing the patch matching metric strategy and using ProtoNet’s matching metric with channel semantic interaction for class prototypes.
(D)	-	✓	✓	Replacing self-supervised pretraining and with supervised pretraining.
(E)	✓	✓	✓	PatSiML.

Note: ✓ means that this method was used in the experiment, otherwise use -.

**Table 8:** Ablation experiments on Mini-ImageNet.

No.	Vit-Small		Swin-Tiny	
	5W1S	5W5S	5W1S	5W5S
A	66.83 ± 0.66	81.96 ± 0.45	67.28 ± 0.67	82.56 ± 0.44
B	69.82 ± 0.65 (↑2.99)	85.33 ± 0.41 (↑3.37)	72.13 ± 0.62 (↑4.85)	85.41 ± 0.41 (↑2.85)
C	68.63 ± 0.66 (↑1.80)	82.87 ± 0.45 (↑0.91)	69.63 ± 0.67 (↑2.35)	83.01 ± 0.44 (↑0.45)
D	52.14 ± 0.60 (↓14.69)	71.40 ± 0.45 (↓10.56)	55.18 ± 0.65 (↓12.10)	67.65 ± 0.45 (↓14.91)
E	**72.26 ± 0.57 (↑5.43)**	**85.39 ± 0.43 (↑3.43)**	**74.33 ± 0.56 (↑7.05)**	**85.89 ± 0.42 (↑3.33)**

Note: The values in the 5W1S and 5W5S columns of the table are accuracy rates and 95% confidence intervals, with values as percentages.

**Table 9:** Ablation experiments on Tiered-ImageNet.

No.	Vit-Small		Swin-Tiny	
	5W1S	5W5S	5W1S	5W5S
A	70.32 ± 0.78	82.35 ± 0.50	70.68 ± 0.71	85.81 ± 0.47
B	74.00 ± 0.73 (↑4.32)	88.26 ± 0.45 (↑2.09)	75.31 ± 0.70 (↑4.63)	87.64 ± 0.49 (↑1.83)
C	71.78 ± 0.71 (↑1.46)	83.54 ± 0.49 (↑1.19)	71.98 ± 0.71 (↑1.30)	86.92 ± 0.47 (↑1.11)
D	59.42 ± 0.65 (↓10.90)	75.34 ± 0.55 (↓7.01)	64.94 ± 0.72 (↓5.74)	77.85 ± 0.45 (↓7.96)
E	**74.74 ± 0.69 (↑4.74)**	**88.90 ± 0.48 (↑6.55)**	**79.56 ± 0.66 (↑8.88)**	**89.51 ± 0.46 (↑3.70)**

Note: The values in the 5W1S and 5W5S columns of the table are accuracy rates and 95% confidence intervals, with values as percentages.

**Table 10:** Ablation experiments of semantic interaction meta-learning methods based on patch matching metric (Tiered-ImageNet).

Back-Bone	Semantic Extractor	Mini-ImageNet		Tiered-ImageNet	
		5W1S	5W5S	5W1S	5W5S
Vit-Small	-	69.82 ± 0.65	85.33 ± 0.41	74.00 ± 0.73	88.26 ± 0.45
	CLIP	**72.26 ± 0.57**	**85.39 ± 0.43**	**74.74 ± 0.69**	**88.90 ± 0.48**
	SBERT	71.96 ± 0.60	85.15 ± 0.49	74.20 ± 0.68	88.76 ± 0.52
	GloVe	71.78 ± 0.59	85.06+0.39	74.68 ± 0.72	88.01 ± 0.51
Swin-Tiny	-	72.13 ± 0.62	85.41 ± 0.41	75.31 ± 0.70	87.64 ± 0.48
	CLIP	**74.33 ± 0.56**	**85.89 ± 0.42**	**79.56 ± 0.66**	**89.50 ± 0.46**
	SBERT	73.60 ± 0.57	84.08 ± 0.44	78.24 ± 0.68	88.97 ± 0.46
	GloVe	72.37 ± 0.60	84.10 ± 0.44	77.73 ± 0.67	89.22 ± 0.44

Note: The values in the 5W1S and 5W5S columns of the table are accuracy rates and 95% confidence intervals, with values in percent.

## Data Availability

Data are contained within the article.

## References

[1] Lai N., Kan M., Han C., Song X., Shan S. (2020). Learning to learn adaptive classifier–predictor for few-shot learning. IEEE Trans. Neural Netw. Learn. Syst..

[2] Doersch C., Gupta A., Zisserman A. (2020). Crosstransformers: Spatially-aware few-shot transfer. Adv. Neural Inf. Process. Syst..

[3] Chen Y., Liu Z., Xu H., Darrell T., Wang X. Meta-baseline: Exploring simple meta-learning for few-shot learning. Proceedings of the IEEE/CVF International Conference on Computer Vision.

[4] Kang S., Hwang D., Eo M., Kim T., Rhee W. Meta-learning with a geometry-adaptive preconditioner. Proceedings of the IEEE/CVF Conference on Computer Vision and Pattern Recognition.

[5] Zhang C., Cai Y., Lin G., Shen C. (2022). Deepemd: Differentiable earth mover’s distance for few-shot learning. IEEE Trans. Pattern Anal. Mach. Intell..

[6] Finn C., Abbeel P., Levine S. Model-agnostic meta-learning for fast adaptation of deep networks. Proceedings of the 34th International Conference on Machine Learning.

[7] Snell J., Swersky K., Zemel R. Prototypical networks for few-shot learning. Proceedings of the Advances in Neural Information Processing Systems 30 (NIPS 2017).

[8] Sung F., Yang Y., Zhang L., Xiang T., Torr P.H., Hospedales T.M. Learning to compare: Relation network for few-shot learning. Proceedings of the IEEE Conference on Computer Vision and Pattern Recognition.

[9] Gidaris S., Komodakis N. Dynamic few-shot visual learning without forgetting. Proceedings of the IEEE Conference on Computer Vision and Pattern Recognition.

[10] Hiller M., Ma R., Harandi M., Drummond T. (2022). Rethinking generalization in few-shot classification. Adv. Neural Inf. Process. Syst..

[11] Chen H., Li H., Li Y., Chen C. (2023). Sparse spatial transformers for few-shot learning. Sci. China Inf. Sci..

[12] Li A., Huang W., Lan X., Feng J., Li Z., Wang L. Boosting few-shot learning with adaptive margin loss. Proceedings of the IEEE/CVF Conference on Computer Vision and Pattern Recognition.

[13] Yang F., Wang R., Chen X. Sega: Semantic guided attention on visual prototype for few-shot learning. Proceedings of the IEEE/CVF Winter Conference on Applications of Computer Vision.

[14] Yan K., Bouraoui Z., Wang P., Jameel S., Schockaert S. Aligning visual prototypes with bert embeddings for few-shot learning. Proceedings of the 2021 International Conference on Multimedia Retrieval.

[15] Xing C., Rostamzadeh N., Oreshkin B., Pinheiro P.O.O. Adaptive cross-modal few-shot learning. Proceedings of the Advances in Neural Information Processing Systems 32 (NeurIPS 2019).

[16] Chen W., Si C., Zhang Z., Wang L., Wang Z., Tan T. Semantic prompt for few-shot image recognition. Proceedings of the IEEE/CVF Conference on Computer Vision and Pattern Recognition.

[17] Zhou J., Wei C., Wang H., Shen W., Xie C., Yuille A., Kong T. (2021). ibot: Image bert pre-training with online tokenizer. arXiv.

[18] Bao H., Dong L., Piao S., Wei F. (2021). Beit: Bert pre-training of image transformers. arXiv.

[19] Ye H.-J., Hu H., Zhan D.-C., Sha F. Few-shot learning via embedding adaptation with set-to-set functions. Proceedings of the IEEE/CVF Conference on Computer Vision and Pattern Recognition.

[20] Hou R., Chang H., Ma B., Shan S., Chen X. Cross attention network for few-shot classification. Proceedings of the Advances in Neural Information Processing Systems 32 (NeurIPS 2019).

[21] Kipf T.N., Welling M. (2016). Semi-supervised classification with graph convolutional networks. arXiv.

[22] Li W., Wang L., Xu J., Huo J., Gao Y., Luo J. Revisiting local descriptor based image-to-class measure for few-shot learning. Proceedings of the IEEE/CVF Conference on Computer Vision and Pattern Recognition.

[23] Radford A., Kim J.W., Hallacy C., Ramesh A., Goh G., Agarwal S., Sastry G., Askell A., Mishkin P., Clark J. Learning transferable visual models from natural language supervision. Proceedings of the 38th International Conference on Machine Learnin.

[24] Vinyals O., Blundell C., Lillicrap T., Wierstra D. Matching networks for one shot learning. Proceedings of the Advances in Neural Information Processing Systems 29 (NIPS 2016).

[25] Ren M., Triantafillou E., Ravi S., Snell J., Swersky K., Tenenbaum J.B., Larochelle H., Zemel R.S. (2018). Meta-learning for semi-supervised few-shot classification. arXiv.

[26] Bertinetto L., Henriques J.F., Torr P.H., Vedaldi A. (2018). Meta-learning with differentiable closed-form solvers. arXiv.

[27] Oreshkin B., López P.R., Lacoste A. Tadam: Task dependent adaptive metric for improved few-shot learning. Proceedings of the Advances in Neural Information Processing Systems 31 (NeurIPS 2018).

[28] Touvron H., Cord M., Douze M., Massa F., Sablayrolles A., Jégou H. Training data-efficient image transformers & distillation through attention. Proceedings of the 38th International Conference on Machine Learning.

[29] Liu Z., Lin Y., Cao Y., Hu H., Wei Y., Zhang Z., Lin S., Guo B. Swin transformer: Hierarchical vision transformer using shifted windows. Proceedings of the IEEE/CVF International Conference on Computer Vision.

[30] Loshchilov I., Hutter F. (2017). Decoupled weight decay regularization. arXiv.

[31] Zhang X., Meng D., Gouk H., Hospedales T.M. Shallow bayesian meta learning for real-world few-shot recognition. Proceedings of the IEEE/CVF International Conference on Computer Vision.

[32] Yang F., Wang R., Chen X. Semantic guided latent parts embedding for few-shot learning. Proceedings of the IEEE/CVF Winter Conference on Applications of Computer Vision.

[33] Hu S.X., Moreno P.G., Xiao Y., Shen X., Obozinski G., Lawrence N.D., Damianou A. (2020). Empirical bayes transductive meta-learning with synthetic gradients. arXiv.

[34] Afrasiyabi A., Lalonde J.-F., Gagné C. (2020). Associative alignment for few-shot image classification. Proceedings of the Computer Vision–ECCV 2020: 16th European Conference.

[35] Dong B., Zhou P., Yan S., Zuo W. (2022). Self-promoted supervision for few-shot transformer. Proceedings of the European Conference on Computer Vision.

[36] Reimers N., Gurevych I. (2019). Sentence-bert: Sentence embeddings using siamese bert-networks. arXiv.

[37] Pennington J., Socher R., Manning C.D. Glove: Global vectors for word representation. Proceedings of the 2014 Conference on Empirical Methods in Natural Language Processing (EMNLP).

